# Massive iron accumulation in PKAN-derived neurons and astrocytes: light on the human pathological phenotype

**DOI:** 10.1038/s41419-022-04626-x

**Published:** 2022-02-25

**Authors:** Paolo Santambrogio, Maddalena Ripamonti, Anna Cozzi, Marzia Raimondi, Chiara Cavestro, Ivano Di Meo, Alicia Rubio, Stefano Taverna, Valeria Tiranti, Sonia Levi

**Affiliations:** 1 IRCCS San Raffaele Scientific Institute, Milan, Italy; 2 Vita-Salute San Raffaele University, Milan, Italy; 3 Medical Genetics and Neurogenetics Unit, Fondazione IRCCS Istituto Neurologico Carlo Besta, Milan, Italy; 4 Institute of Neuroscience, National Research Council, Milan, Italy

**Keywords:** Neurodegeneration, Neurodegeneration

## Abstract

Neurodegeneration associated with defective pantothenate kinase-2 (PKAN) is an early-onset monogenic autosomal-recessive disorder. The hallmark of the disease is the massive accumulation of iron in the *globus pallidus* brain region of patients. PKAN is caused by mutations in the *PANK2* gene encoding the mitochondrial enzyme pantothenate kinase-2, whose function is to catalyze the first reaction of the CoA biosynthetic pathway. To date, the way in which this alteration leads to brain iron accumulation has not been elucidated. Starting from previously obtained hiPS clones, we set up a differentiation protocol able to generate inhibitory neurons. We obtained striatal-like medium spiny neurons composed of approximately 70–80% GABAergic neurons and 10–20% glial cells. Within this mixed population, we detected iron deposition in both PKAN cell types, however, the viability of PKAN GABAergic neurons was strongly affected. CoA treatment was able to reduce cell death and, notably, iron overload. Further differentiation of hiPS clones in a pure population of astrocytes showed particularly evident iron accumulation, with approximately 50% of cells positive for Perls staining. The analysis of these PKAN astrocytes indicated alterations in iron metabolism, mitochondrial morphology, respiratory activity, and oxidative status. Moreover, PKAN astrocytes showed signs of ferroptosis and were prone to developing a stellate phenotype, thus gaining neurotoxic features. This characteristic was confirmed in iPS-derived astrocyte and glutamatergic neuron cocultures, in which PKAN glutamatergic neurons were less viable in the presence of PKAN astrocytes. This newly generated astrocyte model is the first in vitro disease model recapitulating the human phenotype and can be exploited to deeply clarify the pathogenetic mechanisms underlying the disease.

## Introduction

Neurodegeneration associated with deficit of pantothenate kinase-2 (PKAN, OMIM*606157) is a rare monogenic autosomal-recessive disease [1]. It is a member of the neurodegeneration with brain iron accumulation (NBIA) disorders, a group of heterogeneous diseases characterized by common features such as extrapyramidal movements and brain iron accumulation [2, 3]. Usually, PKAN manifests in early childhood with gait disturbances and quickly progresses to a severe movement deficit with dystonia, dysphagia, and dysarthria. The hallmark of this disease is the “eye-of-the-tiger” sign in the *globus pallidus* on T2-weighted magnetic resonance imaging, which reflects the focal accumulation of iron in this area [4]. The disease manifests almost exclusively in the CNS, where a strong reduction in neurons and synapses is evident [5]. There are four *PANK* genes in the human genome encoding PANK enzymes that drive the synthesis of coenzyme A (CoA), a ubiquitous cofactor that activates acyl groups and participates in numerous metabolic processes [6]. Unlike the other isoforms, the PANK2 protein is the only isoform localized in mitochondria [7, 8]. It is mainly expressed in the human brain [9], and it is the most rigorously regulated among all PANK isoforms, being more sensitive to acetyl-CoA inhibition [10].

Several animal models have been developed in an attempt to clarify the molecular and pathological events triggering this disorder: a *Drosophila Pank2* KO model (*fumble*) [11], *pank2* downregulation in zebrafish [12], and *Pank2*-null mice [8, 13]; however, while presenting some pathological features, these animal models lack any evidence of brain iron mishandling, preventing any insight into the causative link between CoA deficiency and brain iron deposition. Recently, *Pank2*-null mice were reconsidered by performing an investigation specifically focused on brain basal ganglia, which revealed perturbation of iron and CoA homeostasis [14]. In addition, a recent report [15] demonstrated that the alteration of CoA biosynthesis led to defects in holo-mtACP complex formation in *Drosophila* and human PKAN cell-line models. Holo-mtACP is the ortholog of human NDUFAB1, a protein with multiple functions: it is involved in Fe–S-cluster biosynthesis [16], lipoylation, activation of pyruvate dehydrogenase, and enhancement of the assembly of respiratory complexes and supercomplexes [17].

Fe–S clusters are cofactors of enzymes involved in many biochemical pathways, including the posttranscriptional regulation of iron-protein expression by IRE/IRP machinery [18]. Thus, a reduced level of holo-mtACP was proposed to elicit iron dyshomeostasis in PKAN pathogenesis. In fact, an in vitro study on *PANK2*-silenced HeLa cells [19], PKAN patient fibroblasts [20, 21], and human-induced pluripotent stem (hiPS)-derived glutamatergic neurons [22, 23] highlighted defective iron metabolism. These findings revealed the association between PANK2 deficiency and impairment of heme and Fe–S-cluster biosynthesis, the two mitochondrial iron-dependent pathways. However, none of these models were characterized by the huge iron deposition typically found in patients’ brains, limiting full comprehension of the human pathogenic mechanism.

Here, we report data obtained from a new mixed neuronal population differentiated from previously developed hiPS clones [22], which are composed mainly of striatal-like medium spiny neurons (derived MSNs, d-MSNs) and, in a small proportion, glial cells. In these PKAN neuronal populations, we detected severe cytosolic iron accumulation, highlighted by Perls staining and ameliorated by CoA treatment. Further analysis specifically targeting astrocytes revealed alterations in iron–protein expression and ferritinophagy [24], as expected in iron-overloaded cells. Excessive iron triggers ferroptosis, which allows PKAN astrocytes to develop a stellate-like reactive phenotype, thus acquiring a cytotoxic feature, confirmed by an increase in glutamate secretion causing reduced survival of PKAN neurons when grown in coculture.

## Results

### Generation and characterization of d-medium spiny neurons

**Fig. 1:**
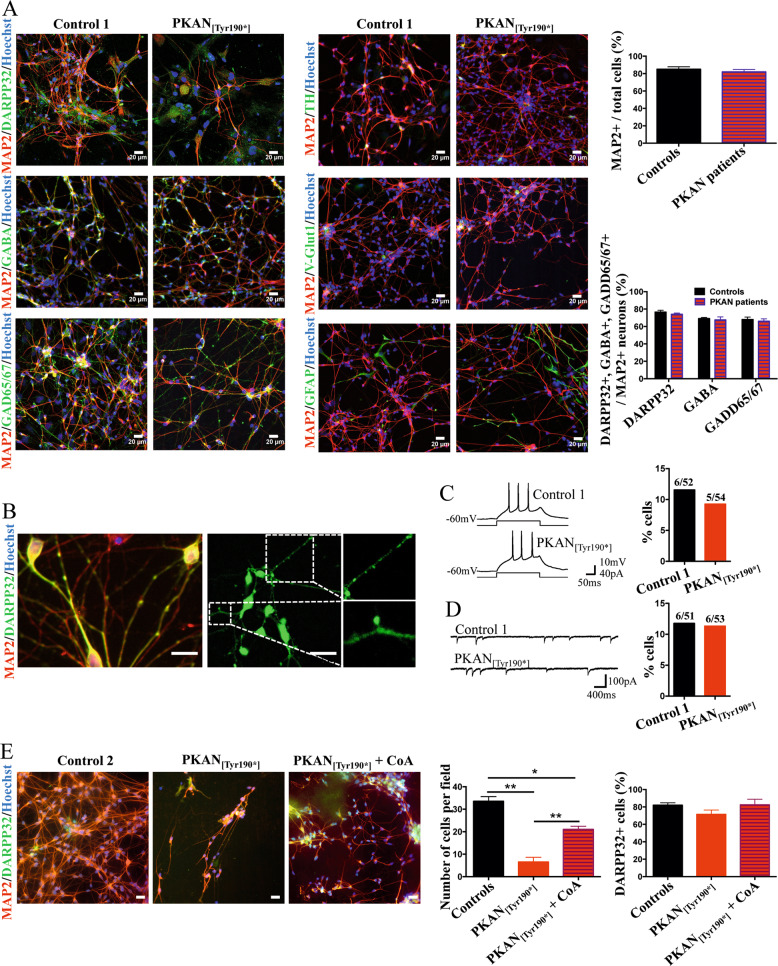
Development and characterization of d-MSNs. **A** Representative immunofluorescence images of d-MSNs from control and patient PKAN_[Tyr190*]_ differentiated for 42 days and stained with the neuronal markers microtubule associated protein 2 (MAP2), dopamine- and cyclic AMP-regulated phosphoprotein 32 (DARPP32), gamma-aminobutyric acid (GABA), glutamic acid decarboxylase 65/67 (GAD65/67), tyrosine hydroxylase (TH), vesicular glutamate transporter 1 (V-Glut1) and the astrocyte marker glial fibrillary acidic protein (GFAP). Nuclei were stained with Hoechst. Scale bars 20 μm. Histograms on the right show the percentage of cells positive for the indicated markers. All data are presented as the mean + SD, *n* = 3. **B** Representative immunofluorescence images of d-MSNs from the control subject stained with MAP2 and DARPP32 with enlargements to highlight the presence of spines in neuronal processes. Scale bars 20 μm. **C** Representative current-clamp recordings showing action potentials elicited in control and PKAN d-MNSs by injection of suprathreshold current steps. The plot on the right represents the fraction of total cells displaying repetitive firing (*p* = 0.7, Z score for 2 population proportions). **D** Examples of spontaneous IPSCs recorded in control and PKAN d-MNSs bathed in extracellular medium containing 5 μM NBQX to block glutamatergic AMPA receptors. Right, summary plot with fractions of total cells displaying IPSCs (*p* = 0.9, Z score for two population proportions). **E** Representative immunofluorescence images of d-MSNs from control and patient PKAN_[Tyr190*]_ differentiated for 42 days in the presence or absence of 20 μm CoA stained with MAP2, DARPP32 and Hoechst. The left histogram shows the number of counted cells per field. Data are presented as the mean + SD, *n* = 3, one-way ANOVA, *p < 0.05, **p < 0.01. The right histogram shows the percentage of cells positive for the DARPP32 marker. Data presented as the mean + SD, *n* = 3.

We used previously obtained hiPS clones [22] of three neonatal normal subjects (referred to herein as controls) and three PKAN patients: one carrying the c. [569_570insA] homozygous mutation of *PANK2* resulting in a protein with a premature stop codon p.[Tyr190*] and two siblings carrying the same c. [1259delG] homozygous mutation that resulted in a protein with a frameshift p.[Gly420Valfs*30]a and b [20, 22]. These mutations led to a complete lack of PANK2 protein in fibroblasts [20]. The control and PKAN hiPS clones were then differentiated into d-MSNs with a method suitable for obtaining inhibitory neurons [25]. At the end of the differentiation protocol after 42 days (Supplementary Fig. 1A), control and PKAN d-MSNs appeared to have developed a dense network and expressed crucial neuronal markers (Fig. 1A). Approximately 80% of the cell population was positive for MAP2 (Fig. 1A), 78% of which were positive for DARPP32 and 70% for GABA and GAD65/67 neuronal markers (Fig. 1A). Only a few cells were TH- or V-glut1-positive (5% and 4%, respectively) (Fig. 1A and Supplementary Fig. 2A), while the number of astrocytes (GFAP+) was variable between experiments, ranging from 10 to 25% (Fig. 1A and Supplementary Fig. 2B). Moreover, bona fide dendritic spines were also visible by MAP2/DARPP32 coimmunostaining (Fig. 1B), indicating the development of a phenotype that recapitulates the main features of native MSNs. Action-potential firing and spontaneous GABAergic synaptic activity were investigated using patch-clamp recordings. Cells (11.5% controls and 9.2% PKAN) responded to injection of suprathreshold current steps with brief action-potential trains (Fig. 1C). Spontaneous inhibitory synaptic currents (sIPSCs) were recorded in 12% control and 11.3% PKAN cells (Fig. 1D). Altogether, these data are suggestive of a similar differentiation of control and PKAN hiPS clones toward a functional MSN phenotype, exhibiting a GABAergic nature. At the ultrastructural level, PKAN d-MSNs showed enlarged and swollen mitochondria with damaged cristae (Supplementary Fig. 2C), thus confirming the data previously obtained on glutamatergic neurons [22].

### PKAN d-MSNs were less viable and accumulated iron, which was partially ameliorated by CoA treatment

**Fig. 2:**
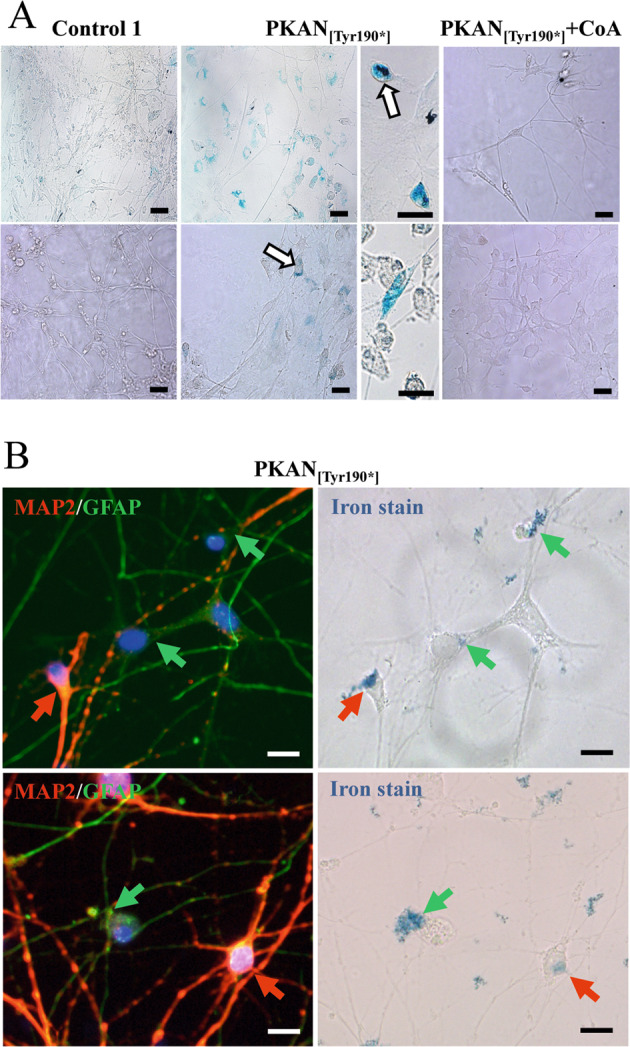
Iron accumulation in the d-MSNs. **A** Representative images of d-MSNs from control and patient PKAN_[Tyr190*]_ differentiated for 42 days in the presence or absence of 20 μm CoA and stained with Perls reaction to view iron granules. White arrows indicate some cells with neuronal shape. Scale bars 20 μm. **B** Representative images of d-MSNs from patient PKAN_[Tyr190*]_ probed after the Perls reaction (iron stain) with anti-MAP2 and GFAP to highlight the cell types positive for iron content. Red and green arrows indicate neurons (MAP2-positive cells) and astrocytes (GFAP-positive cells), respectively. Scale bars 20 μm.

Although the cell-type compositions of d-MSNs derived from PKAN and the control appeared similar, the total number of PKAN cells was considerably reduced with respect to the controls (Fig. 1E). The analysis of the presence of neuronal (MAP2 and DARPP32) and glial (GFAP) markers after 42 days of culture revealed a huge mortality of neurons (Fig. 1A, and E). Notably, the addition of 25 μM CoA during differentiation reduced, at least partially, neuronal mortality (Fig. 1E). This feature did not depend on a reduced ability of patient cells to differentiate into MSNs; in fact, the percentage of DARPP32-positive cells was comparable between CoA-treated and -untreated cells, indicating that CoA supplementation did not affect differentiation (Fig. 1E). In addition, the presence of potential iron load was investigated by the iron-specific Perls staining protocol. Positivity for Perls staining was clearly observed only in PKAN samples (Fig. 2A, central panel). We coupled Perls staining and immunofluorescence using GFAP and MAP2 antibodies to univocally identify iron-positive astrocytes and neurons. Despite the scarcity of surviving neurons, we detected positive Perls-stained cells that colocalized with both neuronal and astrocytic markers (Fig. 2B). This finding implies that the high mortality of neurons might be due to their iron overload or to putative toxicity exerted by iron-loaded astrocytes. Importantly, a consistent reduction in iron-positive cells was observed in PKAN samples exposed to CoA during differentiation (Fig. 2A, right panel), demonstrating that CoA also has a specific effect on iron deposition.

### Generation and characterization of hiPS-derived astrocytes

**Fig. 3:**
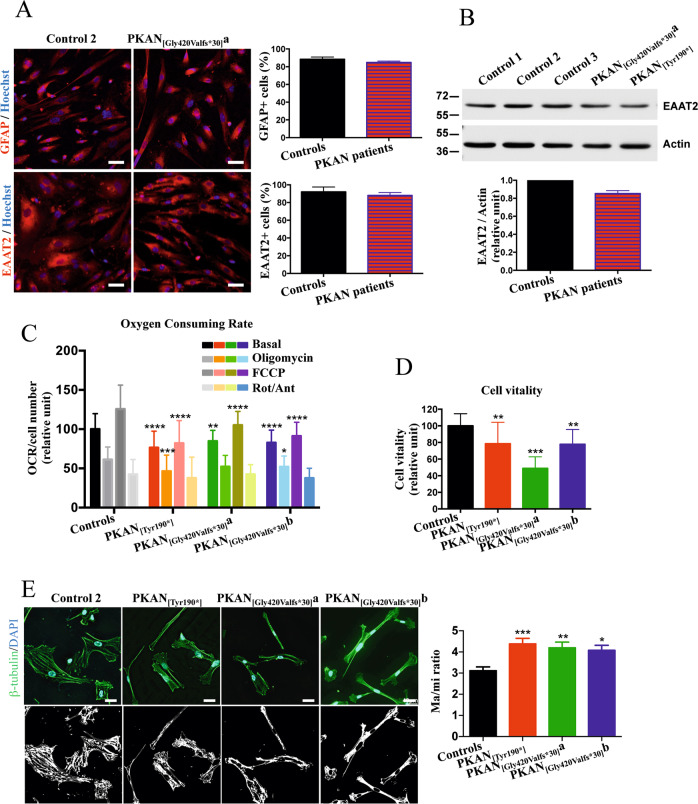
Development and characterization of d-astrocytes. **A** Representative immunofluorescence images of d-astrocytes from control and patient PKAN_[Gly420Valfs*30]_a differentiated for 48 days. Astrocytes were stained with the specific markers glial fibrillary acidic protein (GFAP) and excitatory amino acid transporter 2 (EAAT2). Nuclei were stained with Hoechst. Scale bars 20 μm. Histograms show the percentage of cells positive for the indicated markers. All data are presented as the mean + SD, *n* = 6. **B** Western blot of soluble d-astrocyte homogenates from controls and PKAN patients probed with EAAT2 antibody. Data are representative of three independent experiments. The histogram shows the quantification of EAAT2 normalized to actin by densitometry, *n* = 3. **C** OCR measurements of controls and PKAN d-astrocytes analyzed individually. The plot shows OCR normalization to cell number. OCR was measured under basal conditions and after the addition of oligomycin, carbonyl cyanide 4-trifluoromethoxyphenylhydrazone (FCCP), and rotenone/antimycin A (Rot/Ant) for measurement of, other than basal respiration, ATP-linked respiration, maximal respiratory capacity, and nonmitochondrial respiration, respectively. Bars indicate means + SD, *n* = 3 (two-way ANOVA). *p < 0.05, **p < 0.01, ***p < 0.001, ****p < 0.0001. **D** Histogram shows the cell viability obtained by MTT assay. Bars indicate means + SD, *n* = 3 (one-way ANOVA). **p < 0.01, ***p < 0.001. **E** Representative immunofluorescence images of d-astrocytes from control and PKAN patients stained with the cytoskeletal marker β-tubulin. Nuclei were stained with DAPI (upper panels). In lower panels, the same images are represented as binary threshold images to better visualize cell borders. Scale bars 40μm. The histogram indicates the major/minor-axis (Ma/mi) ratio of individual d-astrocytes. Bars indicate means + SD (one-way Anova). *p < 0.05, ***p < 0.001.

Due to the difficulty in maintaining neurons in culture, we verified whether a pure preparation of astrocytes could be generated and investigated. Control and PKAN hiPS clones were differentiated into neuronal precursor cells (NPCs) [22] and further differentiated into astrocytes (d-astrocytes) (Fig. 3A). Approximately 90% of cells were positive for the specific markers GFAP and EAAT2, demonstrating that this culture was almost exclusively composed of astrocytes (Fig. 3A and B). Respiration was quantified by microscale oxygraphy, which allowed a real-time measurement of global oxygen consumption (Fig. 3C). PKAN d-astrocytes showed significantly lower values of respiratory parameters than controls, suggesting an impairment of mitochondrial functionality (Fig. 3C). We also estimated cell vitality, showing that PKAN d-astrocytes were less viable than controls (Fig. 3D). Given these results, a morphological analysis was performed to evaluate a possible tendency to acquire a stellated phenotype. Classical cultured astrocytes show a flattened/epithelioid phenotype, but in particular conditions, they can reacquire an in vivo star-like aspect through a mechanism named stellation [26]. To quantify potential stellation trends, d-astrocytes were immunolabeled with the specific cytoskeletal marker β-tubulin, and the “stellation tendency” was analyzed (Fig. 3E). PKAN d-astrocytes showed a significantly higher major/minor-axis (Ma/mi) ratio than controls; this condition can be related to a loosening of the classical flattened phenotype in favor of a shrunken phenotype, compatible with a stellated morphology (Fig. 3E).

### PKAN d-astrocytes accumulated iron

**Fig. 4:**
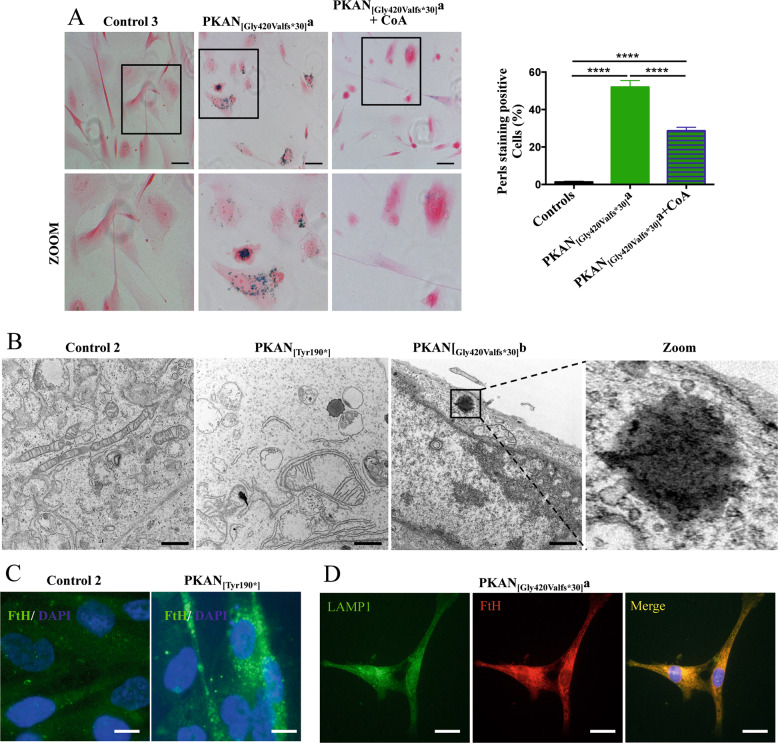
Iron accumulation in the d-astrocytes. **A** Representative images of d-astrocytes from control and patient PKAN_[Gly420Valfs*30]_a, differentiated for 84 days in the presence or absence of 20 μm CoA, stained with the iron-specific Perls reaction (blue), and counterstained with nuclear fast red. Scale bars 20μm. The histogram shows the percentage of cells positive for iron staining. Bars indicate the means + SD of three independent experiments (one-way ANOVA). ****p < 0.0001. **B** Representative images of ultrastructural analyses of d-astrocytes from controls and PKAN patients ([Tyr190*] and [Gly420Valfs*30]b) examined with electron microscopy. Supposed iron aggregate is enlarged in the right panel (Zoom). Scale bars 1 μm. **C** Representative immunofluorescence images of d-astrocytes from control and patient PKAN_[Tyr190*]_ stained with antibodies specific for H-ferritin chains (FtH). Nuclei were stained with DAPI. Scale bars 10 μm. **D** Representative immunofluorescence images of d-astrocytes from patient PKAN_[Gly420Valfs*30]_a stained with the lysosomal marker LAMP1 and antibodies specific for the H-ferritin chain (FtH). Scale bars 20 μm.

Perls reaction showed approximately 50% iron-positive cells in PKAN d-astrocytes and only 1–2% positive cells in controls; this stain appeared with a granular pattern that is characteristic of iron-overloaded cells (Fig. 4A). As in d-MSNs, d-astrocytes were differentiated in the presence of 25 μM CoA, resulting in a consistent reduction in the percentage of Perls-positive cells (Fig. 4A). Ultrastructural analyses of PKAN d-astrocytes revealed the presence of electron-dense granules, reminiscent of the aggregates observed with Perls staining (Fig. 4B Zoom). Furthermore, this analysis confirmed the presence of altered mitochondria, which were enlarged, swollen, and had damaged cristae (Fig. 4B). These findings confirmed the presence of mitochondrial alterations previously observed in GABAergic neurons (Supplemental Fig. 2C) and revealed that electron-dense granules were localized in the cytosol and not in mitochondria.

Usually, iron aggregates colocalize with cytosolic ferritin, the major iron-storage protein, which is targeted to lysosomes for its degradation and iron recycling by a process called ferritinophagy [24]. Specific antibodies against H-chain ferritin (FtH) showed that PKAN d-astrocytes contained higher amounts of ferritin than controls (Fig. 4C). Moreover, a clear colocalization between ferritin and LAMP1, a lysosome marker, emerged, suggesting ferritin-bound iron accumulation in lysosomes (Fig. 4D).

**Fig. 5:**
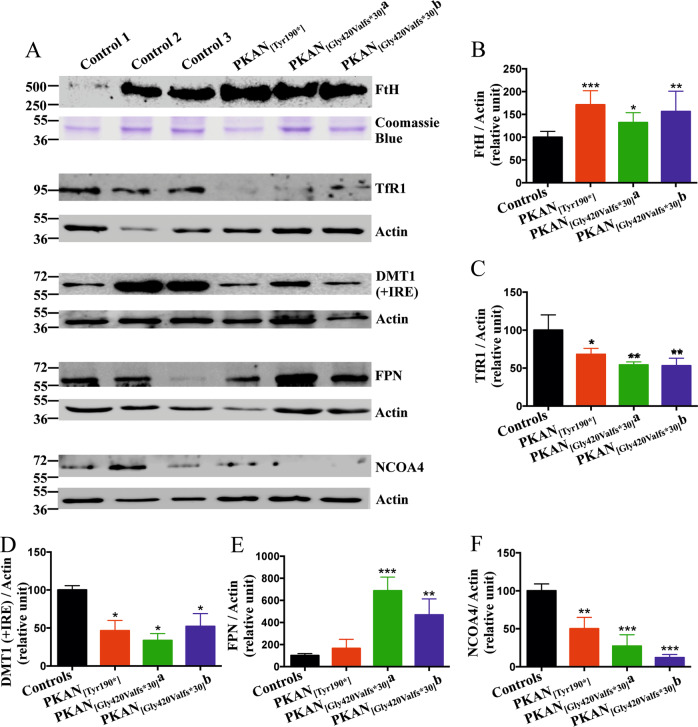
Iron-protein variation in d-astrocytes. **A** Western blot of soluble cell homogenates from d-astrocytes probed with the indicated antibodies. **B–F** Quantification of the indicated proteins normalized to actin by densitometry. Bars indicate means + SD, *n* = 3 (one-way ANOVA). *p < 0.05, **p < 0.01, ***p < 0.001.

The levels of iron-related protein expression were tested by immunoblotting analyses (Fig. 5A). All of these proteins are under the control of the IRE/IRP system, which regulates, at the posttranscriptional level, proteins involved in iron storage (FtH), cellular iron uptake (TfR1 and DMT1) and iron release (FPN). In addition, nuclear receptor coactivator 4 (NCOA4), the carrier protein for ferritin lysosomal degradation, was evaluated (Fig. 5A). The data revealed increased contents of FtH and FPN and reduced contents of TfR1, DMT1 ( + IRE) and NCOA4 in PKAN d-astrocytes compared with controls, indicating that the machinery regulating iron homeostasis responded to an iron-accumulation scenario (Fig. 5 B–F).

### PKAN d-astrocytes are subjected to ferroptosis

**Fig. 6:**
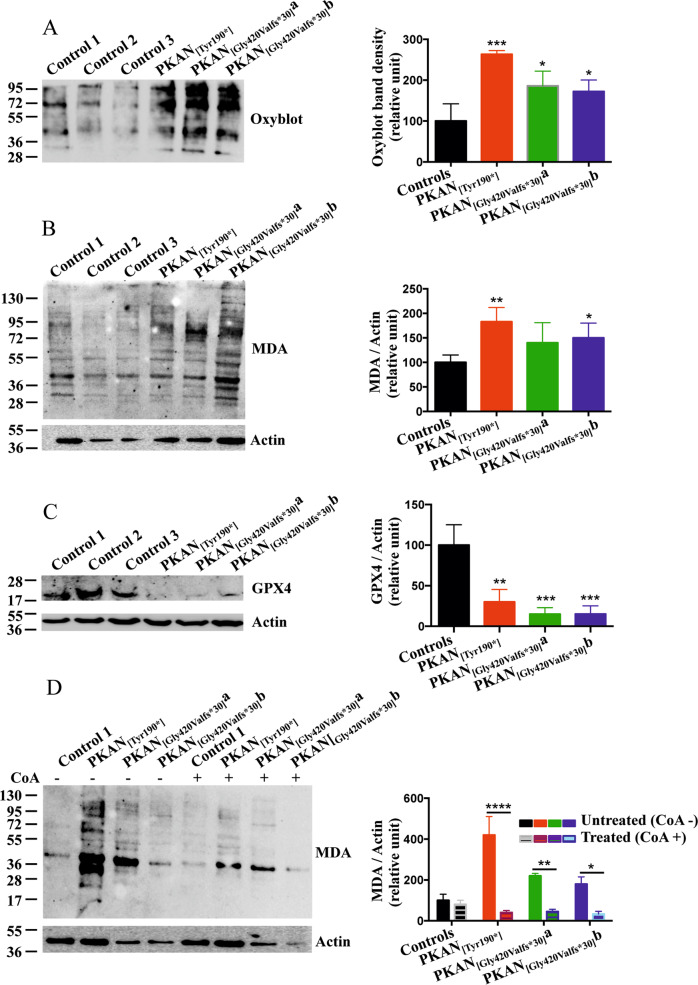
Alteration of oxidative status and ferroptosis in d-astrocytes. **A** Representative images of Oxyblot analysis of soluble cell homogenates from d-astrocytes. The histogram shows the levels of carbonylated proteins quantified by densitometry. Bars indicate means + SD, *n* = 3 (one-way ANOVA). *p < 0.05, ***p < 0.001. **B** Western blot of soluble cell homogenates from d-astrocytes probed for lipoperoxidation with anti-MDA. The histogram shows the levels of lipid peroxidation quantified by densitometry. Bars indicate means + SD, *n* = 3 (one-way ANOVA). *p < 0.05, **p < 0.01. **C** Western blot of soluble cell homogenates from d-astrocytes probed with anti-GPX4. The histogram shows the levels of the protein quantified by densitometry. Bars indicate means + SD, *n* = 3 (one-way ANOVA). **p < 0.01, ***p < 0.001. **D** Western blot of soluble cell homogenates from d-astrocytes differentiated in the presence or absence of 20 μM CoA and probed for lipoperoxidation with anti-MDA. The histogram shows the levels of lipid peroxidation quantified by densitometry. Bars indicate means + SD, *n* = 3 (one-way ANOVA). *p < 0.05, **p < 0.01, ****p < 0.0001.

The iron load detected in PKAN d-astrocytes suggests the possibility of alteration of cell oxidative status and consequent cell death through a mechanism known as ferroptosis, which is an iron-dependent lipid-peroxidation-driven cell death cascade [27]. The presence of higher amounts of oxidized proteins in PKAN astrocytes was detected by oxyblot (Fig. 6A). Then, the presence of ferroptosis signs was revealed by monitoring the amount of malondialdehyde (MDA), a marker of lipid peroxidation (Fig. 6B), and the level of glutathione peroxidase 4 (GPX4), an enzyme that protects cells from lipid peroxidation (Fig. 6C). The results showed a higher level of lipid peroxidation and a lower amount of GPX4 in PKAN d-astrocytes (Fig. 6B and C), indicating signs of ferroptosis. Interestingly, the presence of 25 μM CoA during astrocyte differentiation led to a decreased level of MDA in PKAN d-astrocytes, eventually preventing the ferroptotic phenotype (Fig. 6D).

### PKAN d-astrocytes exert cytotoxic effects on glutamatergic neurons

**Fig. 7:**
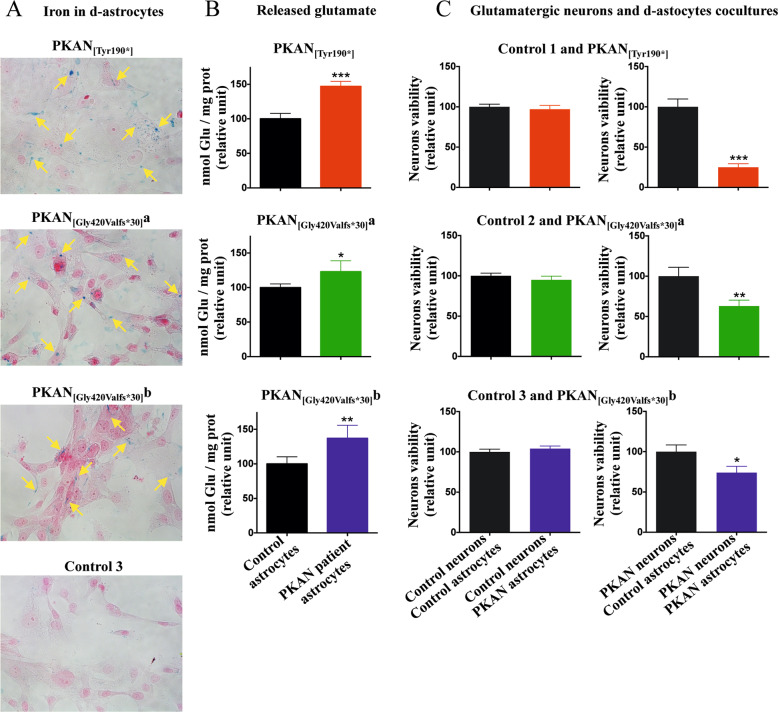
Cytotoxic effect of d-astrocytes on glutamatergic neurons. **A** Representative images of d-astrocytes from control and PKAN patients stained with the iron-specific Perls reaction (blue) and counterstained with nuclear fast red. Yellow arrows indicate iron granules. **B** Amount of glutamate released by the d-astrocytes measured by glutamate assay kit colorimetric (ABCAM) normalized on cell total protein content. Bars indicate means + SD, *n* = 3 (Student’s *t-*test). *p < 0.05, **p < 0.01, ***p < 0.001. **C** Mature glutamatergic neurons and d-astrocytes were cocultured for three days, and after removal of the d-astrocytes, the glutamatergic neurons were quantified by measuring the DAPI staining using a Tecan plate reader. Bars indicate means + SD, *n* = 3 (Student’s *t-*test). *p < 0.05, **p < 0.01, ***p < 0.001.

To test the effect of PKAN d-astrocytes on neurons, we cocultured hiPS-derived glutamatergic neurons and d-astrocytes for three days. We chose a glutamatergic phenotype due to the difficulty of obtaining pure hiPS-derived GABAergic neurons. The d-astrocytes used for the coculture were also subjected to Perls staining, and the results indicated that the majority of PKAN d-astrocytes were in a massive iron-overload condition (Fig. 7A). Moreover, these d-astrocytes showed a higher release of glutamate (Fig. 7B), which could lead to a potential deadly excitotoxic effect on neurons. The amount of nuclear DAPI staining, quantified after the removal of the inserts containing d-astrocytes, was exploited as a surrogate marker of neuron survival. In the short time used for coculture, neither control nor PKAN glutamatergic neuron vitality was affected by the presence of control d-astrocytes (Fig. 7C). Conversely, while PKAN glutamatergic neurons were less viable when cocultured with PKAN d-astrocytes, control glutamatergic neurons were not affected by the presence of PKAN d-astrocytes (Fig. 7C), suggesting that the neurotoxic behavior of PKAN astrocytes was particularly effective on vulnerable PKAN glutamatergic neurons.

## Discussion

Although iron accumulation is the hallmark of PKAN, its relationship with dysfunctional CoA biosynthesis is not completely clear. CoA and CoA derivatives are crucial substrates for ATP generation via the tricarboxylic acid cycle, fatty acid metabolism, cholesterol and ketone-body biosynthesis, and histone and nonhistone protein acetylation [28]. Moreover, few proteins need to be activated by the transfer of a 4′-phosphopantetheine moiety from CoA. Among these, the *E. coli* mitochondrial acyl carrier protein (mtACP*Ec*, NDUFAB1 in humans) needs 4′-phosphopantetheinylation to be functional. This protein is involved in fatty acid synthesis and is a component of the human Fe–S biogenesis complex [29]. This finding suggested that mtACP may connect fatty acid metabolism to Fe–S-cluster biosynthesis [30], allowing to hypothesize that the lack of 4′-phosphopantetheinylation of mtACP could be responsible for iron impairment in some PKAN models [15]. Nevertheless, these processes are vital for any cell type, and it remains unexplained why the disease primarily affects the central nervous system and why iron accumulates exclusively in the brain. Our incomplete knowledge is essentially due to the lack of models that develop iron overload. To overcome this limitation, we generated a novel neuronal population of d-MSNs that better mimicked neuron degeneration in the disease than previously studied glutamatergic neurons [22, 23].

We obtained a mixed neuronal population of GABAergic neurons and astrocytes characterized by iron deposition. To our knowledge, this is the first human neuronal model recapitulating the typical hallmark of the disorder, thus representing a suitable cellular system in which pathological phenotypes are investigated.

Although iron aggregates characterize both d-MSNs and d-astrocytes, the latter survived longer than d-MSNs, highlighting a major capacity to counteract iron-overload effects. This in vitro finding is in agreement with the histopathological data described in autoptic samples from the *globus pallidus* of six PKAN patients [5], where Perls staining detected the presence of iron, particularly evident in the cytoplasm of astrocytes but also in some neurons. Moreover, ferritin immunostaining partially coincided with Perls staining and was significantly increased in astrocytes [5].

**Fig. 8:**
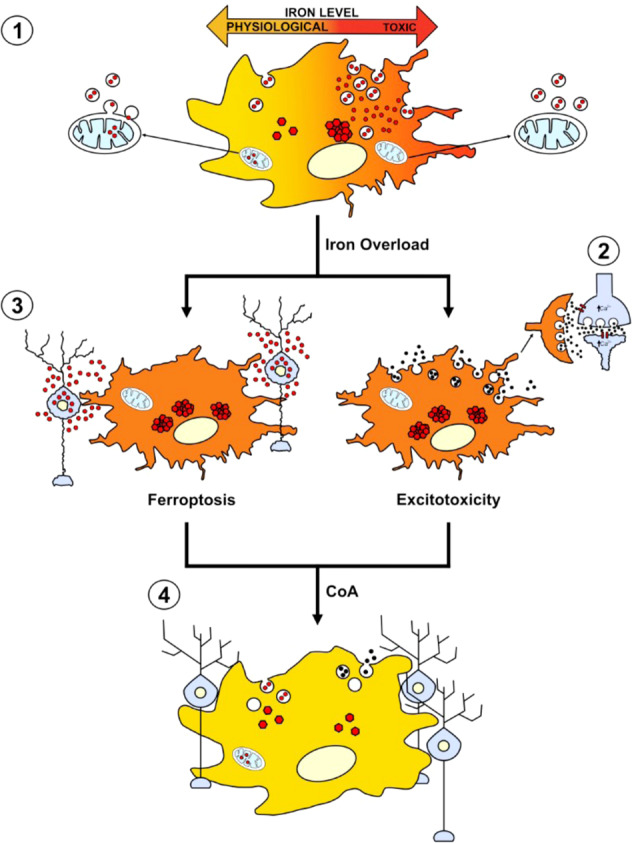
Cartoon of hypothetical mechanism of PKAN neuronal death. The alteration of endosomal trafficking due to CoA deficiency leads to inefficient iron transport to mitochondria and cytosolic iron excess. Subsequent neurodegeneration occurs by excitotoxicity and aberrant release of toxic amounts of gliotransmitters, such as glutamate (2), or by ferroptosis (3), from reactive astrocytes. Treatment with CoA rescues CoA deficiency-associated impairment of endosomal trafficking (4), thus impeding iron overload and neuronal death.

The picture in our PKAN d-astrocyte model appeared similar since iron deposition was mainly associated with cytosolic ferritin [18]. In contrast, PKAN d-astrocytes did not show evidence of iron accumulation in mitochondria that, although morphologically altered, did not contain electron-dense aggregates in EM analysis. Nevertheless, respiratory activity was reduced, confirming data previously obtained on PKAN glutamatergic neurons where a general impairment of mitochondrial iron-dependent biosynthesis was detected [22], indicating the inability of mitochondria to properly manage iron. In PKAN d-astrocytes, cytosolic iron accumulation, confirmed by the variable expression of iron-dependent proteins, suggested that the physiological flux of iron into mitochondria was impaired. This might be explained by the effect of CoA deficiency on membrane integrity, which is needed to maintain the correct delivery of iron to mitochondria (Fig. 8). One route of mitochondrial iron intake, demonstrated until now in reticulocytes, is the so-called “Kiss and Run” pathway, which involves the association of holo-transferrin-loaded endosomes with mitochondria [31, 32]. A more conceivable explanation for the impairment of endosomal trafficking is the alteration of CoA-dependent palmitoylation of TfR1, which was described to be altered in PKAN fibroblasts [21] and causative of iron overload in Friedreich ataxia fibroblasts [33].

However, we cannot exclude that the impairment of iron intake in the mitochondria derives from cellular energy deficiency as a consequence of the lack of NDUFAB1 acetylation, which is also a component of complex I of the respiratory chain in mammals [34]. Further experiments are needed to clarify this issue.

In the cytosolic compartment, a *de novo* machinery for Fe–S-cluster biogenesis exists [16]. Thus, the presence of the Fe–S-cluster may properly regulate the IRE/IRP machinery, which senses cytosolic redox-active iron excess and stimulates the posttranscriptional regulation of iron-related protein expression [18], leading to an appropriate reaction to cellular iron loading. It should be noted that CoA addition in d-astrocyte culture was able to limit the process of cytosolic iron aggregation, confirming the interrelationship between CoA deficiency and iron dyshomeostasis (Fig. 8). This evidence is particularly significant from a therapeutic point of view. In recent years, considerable effort has been made to set up preclinical or clinical trials aimed at reconstitution of CoA levels or reduction of brain iron deposition (reviewed in [35]). However, disease-modifying therapies targeting the pathogenic mechanism and able to counteract disease progression are still lacking. Our data clearly established the efficacy of CoA treatment in vitro to ameliorate pathologic events, including iron deposition, and support the current hypothesis establishing, for metal overload, a subordinate effect on altered CoA metabolism in disease progression. However, excess redox-active iron triggers alteration of oxidative status, preparing PKAN d-astrocytes for ferroptosis, and enhancing their propensity to move toward a stellation process (Fig. 8). Stellation is described as a reactive phenotype induced in 2D cultured astrocytes by, among other factors, inhibitors of Rho kinase (ROCK), resulting in deep cellular changes, including an increase in L-glutamate secretion and enhanced expression of antioxidant genes [36]. Our neuron–astrocyte coculture experiments indicated that PKAN d-astrocytes acquired neurotoxic properties related to a higher level of glutamate secretion, suggesting a major role for these cells in causing neuronal death (Fig. 8). Further investigations are needed to establish whether this neurotoxic attitude is a cause or an effect of iron overload and if it elicits a state of reactive gliosis. This could definitively clarify the role of iron in PKAN pathogenesis and help with the design of a novel optimized therapy.

## Materials and methods

All methods have related literature references. All d-MSNs and d-astrocytes from controls and PKAN patients were subjected to the same analyses, with one example reported in each figure. Further protocol information is available from the corresponding author upon reasonable request.

### Generation of hiPSCs and striatal-like medium spiny neurons

Independent hiPS clones for each control and PKAN patient were previously generated and fully characterized [22]. The d-MSNs were obtained following the procedure previously described [25, 37] and illustrated in Supplementary Fig. 1.

### Generation of human astrocytes from NPC

The same hiPS clones from control and PKAN patients were differentiated into a pure and stable population of self-renewable NPCs as in [22]. NPCs were seeded onto Matrigel-coated plates and grown in DMEM-F12 supplemented with 1% Pen/Strep (Sigma), 2 mM L-glutamine (Sigma), N2 (1:100, Life Technologies), B27 (1:200, Life Technologies), and bFGF (20 ng/ml, Tebu-Bio). After reaching 60% confluence, the medium was supplemented by the addition of 20% FBS, and the culture was maintained for more than 45 days to produce astrocytes with a good level of maturation.

### Coculture of cells in the Transwell plate system

NPCs from controls and PKAN patients were seeded on Matrigel-coated 24-well plates and cultured in differentiating neuronal medium for 3 weeks to develop a population of glutamatergic neurons [22]. Astrocytes, produced as described above, were seeded on a 24-well cell culture insert (5 × 10^5^ cells/insert). The inserts with astrocytes were then added to the plate with neurons, and the coculture was maintained in neuronal medium with 5% dialysate serum for 3 days. Inserts were removed, and the neurons were fixed in 4% paraformaldehyde, permeabilized with 0.1% Triton X-100, and the nuclei were stained with DAPI (1.5 μg/ml). DAPI fluorescence was quantified by a Tecan plate reader at 367/452 nm excitation/emission wavelengths.

### Cell viability assay

A total of 2 × 10^4^ cells/well in 96-well plates were grown in the appropriate medium at 37 °C for 18 h and then incubated with 10 μl of MTT solution (5 mg/ml in phosphate-buffered saline) (Sigma-Aldrich) for 2 h at 37 °C. The color absorbance was read at 570 nm.

### Immunofluorescence, immunoblotting, and oxidized protein detection

Cells grown on coverslips were fixed in 4% paraformaldehyde and processed for immunofluorescence as described in [38]. Specific antibodies and conditions are listed in Supplementary Table 1. Images were acquired by a Zeiss Axio Observer Z1 fluorescence microscope equipped with a Hamamatsu EM-CCD 9100-02 camera and Volocity acquisition software. Immunoblotting was performed after separation of soluble proteins (20 μg) by SDS-PAGE as in Orellana [22]. The signal was revealed using an ECL kit (GE Healthcare) and detected with a ChemiDoc MP Imaging System (BIORAD). Oxidized proteins were detected using the Oxyblot Protein Oxidation Detection Kit (Millipore) as described in [39]. Total protein contents were measured using the BCA protein assay (Pierce) calibrated with bovine serum albumin.

### Determination of respiratory activity

The oxygen-consumption rate (OCR) was measured in PKAN and control astrocytes with an XF96 Extracellular Flux Analyzer (Seahorse Bioscience, Billerica, MA, USA) as described previously [22].

### Measurement of released glutamate

Mature astrocytes were seeded onto Matrigel-coated plates and grown in astrocyte medium supplemented with 20% dialyzed FBS. Astrocytes were incubated in medium without FBS for 1 h prior to the glutamate test. The amount of glutamate secreted in the medium was determined using the Glutamate Assay Kit Colorimetric (Abcam). Astrocytes were then washed with PBS and lysed in 20 mM Tris-HCl, pH 7.4, and 1% Triton X-100. The total protein content was measured in clear supernatants and used to normalize the amount of glutamate.

### Electrophysiology

Inhibitory post synaptic currents (IPSCs) and action potentials were recorded in d-MSNs at day 42 from the beginning of differentiation. Recordings were performed in whole-cell configuration as in [22]. Data acquisition and analyses were performed using pClamp software (Molecular Devices).

### Morphological analyses

For morphological investigations, cells were fixed and stained with the specific cytoskeletal marker β-tubulin. Fluorescence images were acquired with a GE healthcare DeltaVision™ Ultra or Leica SP5 laser-scanning confocal microscope equipped with proper excitation/emission filters. Analyses for stellation tendency were run on fluorescence images as follows: major (Ma) and minor (mi) axes were measured for each entire and discernible astrocyte present in the acquired field in controls and PKAN samples, and the Ma/mi ratio was calculated as an index of the classical flat, epithelioid phenotype lost.

### Electron microscopy

d-MSNs and d-astrocytes were fixed in 4% paraformaldehyde and 2.5% glutaraldehyde, postfixed with 2% OsO4, washed, dehydrated, and embedded in Epon 812. Thin sections were stained with uranyl acetate and lead citrate and examined under an EFTEM Leo912 electron microscope (Zeiss, Milano, Italy). Images were randomly obtained under blind conditions for the examiner.

### Statistical analyses

Statistical methods to predetermine the sample size in the experiments were not employed. All experiments were performed at least in triplicate, and the data were analyzed using GraphPad Prism. In general, the normally distributed data were analyzed using two-tailed unpaired Student’s *t*-test and one- or two-way ANOVA followed by the Bonferroni post test. All data are reported as the mean +SD. A *p*-value <0.05 was used to determine significance.

## Supplementary information

Supplementary material

English Editing Certificate

Checklist

## Data Availability

The datasets generated and/or analyzed during the current study are available from the corresponding authors on reasonable request.

## References

[1] Zhou B, Westaway SK, Levinson B, Johnson MA, Gitschier J, Hayflick SJ. A novel pantothenate kinase gene (PANK2) is defective in Hallervorden-Spatz syndrome. Nat Genet [Internet]. 2001;28:345–9. https://pubmed.ncbi.nlm.nih.gov/11479594/. 10.1038/ng572 11479594

[2] Levi S, Tiranti V (2019). Neurodegeneration with brain iron accumulation disorders: Valuable models aimed at understanding the pathogenesis of Iron deposition. Pharmaceuticals.

[3] Kurian MA, Hayflick SJ (2013). Pantothenate kinase-associated neurodegeneration (PKAN) and PLA2G6-associated neurodegeneration (PLAN): review of two major neurodegeneration with brain iron accumulation (NBIA) phenotypes. Int Rev Neurobiol.

[4] Zorzi G, Zibordi F, Chiapparini L, Bertini E, Russo L, Piga A (2011). Iron-related MRI images in patients with pantothenate kinase-associated neurodegeneration (PKAN) treated with deferiprone: results of a phase II pilot trial. Mov Disord.

[5] Kruer MC, Hiken M, Gregory A, Malandrini A, Clark D, Hogarth P (2011). Novel histopathologic findings in molecularly-confirmed pantothenate kinase-associated neurodegeneration. Brain.

[6] Dansie LE, Reeves S, Miller K, Zano SP, Frank M, Pate C (2014). Physiological roles of the pantothenate kinases. Biochem Soc Trans.

[7] Johnson MA, Kuo YM, Westaway SK, Parker SM, Ching KHL, Gitschier J, et al. Mitochondrial localization of human PANK2 and hypotheses of secondary iron accumulation in pantothenate kinase-associated neurodegeneration. Ann N Y Acad Sci [Internet]. 2004;1012:282–98. https://pubmed.ncbi.nlm.nih.gov/15105273/. 10.1196/annals.1306.023 15105273

[8] Brunetti D, Dusi S, Morbin M, Uggetti A, Moda F, D'Amato I (2012). Pantothenate kinase-associated neurodegeneration: altered mitochondria membrane potential and defective respiration in pank2 knock-out mouse model. Hum Mol Genet.

[9] Hayflick SJ, Westaway SK, Levinson B, Zhou B, Johnson MA, Ching KHL, et al. Genetic, clinical, and radiographic delineation of Hallervorden-Spatz syndrome. JN Engl J Med [Internet]. 2003;348:33–40. https://pubmed.ncbi.nlm.nih.gov/12510040/. 10.1056/NEJMoa020817 12510040

[10] Garcia M, Leonardi R, Zhang Y-M, Rehg JE, Jackowski S (2012). Germline deletion of pantothenate kinases 1 and 2 reveals the key roles for CoA in postnatal metabolism. PLoS ONE.

[11] Yang Y, Wu Z, Kuo YM, Zhou B (2005). Dietary rescue of fumble-a Drosophila model for pantothenate-kinase-associated neurodegeneration. J Inherit Metab Dis.

[12] Zizioli D, Tiso N, Guglielmi A, Saraceno C, Busolin G, Giuliani R (2016). Knock-down of pantothenate kinase 2 severely affects the development of the nervous and vascular system in zebrafish, providing new insights into PKAN disease. Neurobiol Dis.

[13] Brunetti D, Dusi S, Giordano C, Lamperti C, Morbin M, Fugnanesi V (2014). Pantethine treatment is effective in recovering the disease phenotype induced by ketogenic diet in a pantothenate kinase-associated neurodegeneration mouse model. Brain.

[14] Jeong SY, Hogarth P, Placzek A, Gregory AM, Fox R, Zhen D (2019). 4'-Phosphopantetheine corrects CoA, iron, and dopamine metabolic defects in mammalian models of PKAN. EMBO Mol Med.

[15] Lambrechts RA, Schepers H, Yu Y, van der Zwaag M, Autio KJ, Vieira-Lara MA (2019). CoA-dependent activation of mitochondrial acyl carrier protein links four neurodegenerative diseases. EMBO Mol Med.

[16] Maio N, Rouault TA (2020). Outlining the complex pathway of mammalian Fe-S cluster biogenesis. Trends Biochem Sci.

[17] Zhang R, Hou T, Cheng H, Wang X (2019). NDUFAB1 protects against obesity and insulin resistance by enhancing mitochondrial metabolism. FASEB J.

[18] Hentze MW, Muckenthaler MU, Galy B, Camaschella C (2010). Two to Tango: regulation of mammalian iron metabolism. Cell.

[19] Poli M, Derosas M, Luscieti S, Cavadini P, Campanella A, Verardi R (2010). Pantothenate kinase-2 (Pank2) silencing causes cell growth reduction, cell-specific ferroportin upregulation and iron deregulation. Neurobiol Dis.

[20] Santambrogio P, Dusi S, Guaraldo M, Rotundo LI, Broccoli V, Garavaglia B (2015). Mitochondrial iron and energetic dysfunction distinguish fibroblasts and induced neurons from pantothenate kinase-associated neurodegeneration patients. Neurobiol Dis.

[21] Drecourt A, Babdor J, Dussiot M, Petit F, Goudin N, Garfa-Traore M (2018). Impaired transferrin receptor palmitoylation and recycling in neurodegeneration with brain iron accumulation. Am J Hum Genet.

[22] Orellana DI, Santambrogio P, Rubio A, Yekhlef L, Cancellieri C, Dusi S (2016). Coenzyme A corrects pathological defects in human neurons of PANK2-associated neurodegeneration. EMBO Mol Med.

[23] Arber C, Angelova PR, Wiethoff S, Tsuchiya Y, Mazzacuva F, Preza E (2017). iPSC-derived neuronal models of PANK2-associated neurodegeneration reveal mitochondrial dysfunction contributing to early disease. PLoS ONE.

[24] Mancias JD, Wang X, Gygi SP, Harper JW, Kimmelman AC (2014). Quantitative proteomics identifies NCOA4 as the cargo receptor mediating ferritinophagy. Nature.

[25] Arber C, Precious SV, Cambray S, Risner-Janiczek JR, Kelly C, Noakes Z (2015). Activin a directs striatal projection neuron differentiation of human pluripotent stem cells. Development.

[26] Racchetti G, Alessandro RD, Meldolesi J (2012). Astrocyte stellation, a process dependent on Rac1 is sustained by the regulated exocytosis of enlargeosomes. Glia.

[27] Dixon SJ, Lemberg KM, Lamprecht MR, Skouta R, Eleina M, Gleason CE (2013). NIH Public Access.

[28] Siudeja K, Srinivasan B, Xu L, Rana A, de Jong J, Nollen EAA. et al.Impaired Coenzyme A metabolism affects histone and tubulin acetylation in Drosophila and human cell models of pantothenate kinase associated neurodegeneration. EMBO Mol Med. 2011;3:755–66. https://pubmed.ncbi.nlm.nih.gov/21998097/. 10.1002/emmm.201100180 PMC3377114 21998097

[29] Cai K, Tonelli M, Frederick RO, Markley JL. Human Mitochondrial Ferredoxin 1 (FDX1) and Ferredoxin 2 (FDX2) Both Bind Cysteine Desulfurase and Donate Electrons for Iron-Sulfur Cluster Biosynthesis. Biochemistry [Internet]. 2017;56:487–99. https://pubmed.ncbi.nlm.nih.gov/28001042/. 10.1021/acs.biochem.6b00447 PMC5267338 28001042

[30] Van Vranken JG, Jeong MY, Wei P, Chen YC, Gygi SP, Winge DR (2016). The mitochondrial acyl carrier protein (ACP) coordinates mitochondrial fatty acid synthesis with iron sulfur cluster biogenesis. Elife.

[31] Hamdi A, Roshan TM, Kahawita TM, Mason AB, Sheftel AD, Ponka P. Erythroid cell mitochondria receive endosomal iron by a “kiss-and-run” mechanism. Biochim Biophys Acta - Mol Cell Res [Internet]. 2016;1863:2859–67. 10.1016/j.bbamcr.2016.09.008. 10.1016/j.bbamcr.2016.09.008 27627839

[32] Das A, Nag S, Mason AB, Barroso MM (2016). Endosome-mitochondria interactions are modulated by iron release from transferrin. J Cell Biol.

[33] Petit F, Drecourt A, Dussiot M, Zangarelli C, Hermine O, Munnich A (2021). Defective palmitoylation of transferrin receptor triggers iron overload in Friedreich ataxia fibroblasts. Blood.

[34] Dibley MG, Formosa LE, Lyu B, Reljic B, McGann D, Muellner-Wong L (2020). The mitochondrial acyl-carrier protein interaction network highlights important roles for LYRM family members in complex i and mitoribosome assembly. Mol Cell Proteom.

[35] Thakur N, Klopstock T, Jackowski S, Kuscer E, Tricta F, Videnovic A (2021). Rational design of novel therapies for pantothenate kinase–associated neurodegeneration. Mov Disord.

[36] O'Shea RD, Lau CL, Zulaziz N, Maclean FL, Nisbet DR, Horne MK (2015). Transcriptomic analysis and 3D bioengineering of astrocytes indicate ROCK inhibition produces cytotrophic astrogliosis. Front Neurosci.

[37] Iannielli A, Ugolini GS, Cordiglieri C, Bido S, Rubio A, Colasante G (2019). Reconstitution of the human nigro-striatal pathway on-a-chip reveals OPA1-dependent mitochondrial defects and loss of dopaminergic synapses. Cell Rep.

[38] Santambrogio P, Ripamonti M, Paolizzi C, Panteghini C, Carecchio M, Chiapparini L (2020). Harmful iron-calcium relationship in pantothenate kinase associated neurodegeneration. Int J Mol Sci.

[39] Cozzi A, Orellana DI, Santambrogio P, Rubio A, Cancellieri C, Giannelli S (2019). Stem cell modeling of neuroferritinopathy reveals iron as a determinant of senescence and ferroptosis during neuronal aging. Stem Cell Reports.

