# Supplementary material for: Massive iron accumulation in PKAN-derived neurons and astrocytes: light on the human pathological phenotype
Source: Cell Death Dis. 2022 Feb 25;13(2):185. doi: 10.1038/s41419-022-04626-x (PMC8881507; doi:10.1038/s41419-022-04626-x)
Supplement: Supplementary file 1 — Supplementary material [file 41419_2022_4626_MOESM1_ESM.docx]

**Supplementary Figure 1. Schematic representation and detailed procedure used to differentiate d-MSN.** The scheme describes the passage used to obtain mature d-MSN. Days of culture and time and length of small molecules added are indicated. hiPSCs were seeded onto plates coated with Matrigel (Corning) in StemFlex basal medium plus StemFlex supplement (Gibco) containing 1% penicillin/streptomycin (Lonza). When they reach a confluence of about 70% the medium was changed with DMEM-F12/Neurobasal media (2:1) supplemented with 1% Pen/Strep, 2mM L-glutamine (Sigma), N2 (1:100, Life Technologies), B27 (1:50, Life Technologies), SB431542 (10μM, tebu-bio), LDN-193189 (100nM, Sigma), dorsomorphin (200nM, DBA). Medium was changed every 2-3-days. From day 5 the same medium without SB431542 was used. At day 9 cells were detached with accutase and plated 1:3 avoiding single cell suspension onto matrigel coated plates in DMEM-F12/Neurobasal media (2:1) supplemented with 1% Pen/Strep, 2mM L-glutamine, N2, B27, activin A (25ng/ml, R&D Systems). Medium was changed every 2-3-days. Next passages were performed with accutase and plated as single cell suspension. At day 20 the presence of NPCs were checked by immunofluorescence. From day 25 BDNF (10ng/ml, Peprotech) and GDNF (10ng/ml, Peprotech) were added to the medium to drive differentiation toward MSNs. ­­­

*Striatal-like Medium Spiny Neurons*

(d-MSNs)

hiPSCs


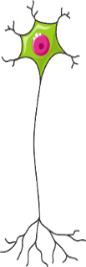

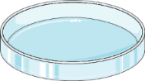

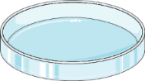

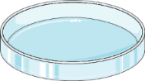

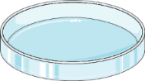

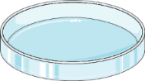


Day 0

Day 5

Day 9

Day 18

Day 25

Day 42

SB431542, LDN, Dorsomorphin

LDN, Dorsomorphin

Activin A

P1

P2

Activin A

Activin A, BDNF, GDNF


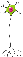

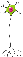

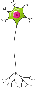


C

**TH**

**V-Glut1**

**TH+, V-Glut1+**

**/ MAP2+ neurons (%)**

**Map2**

**GFAP**

**MAP2+, GFAP+**

**/ total cells (%)**

A

B

**Supplementary Figure 2. Characterization of d-MSN. (A and B)** Histograms with the percentage of cells positive for the indicated markers obtained from the experiment showed in Fig. 1. All data are presented as the mean + SD. Plot A *n* = 3, plot B *n* = 4. (**C**) Representative images of ultrastructural analyses of fixed d-MSN from controls and PKAN patients examined with electron microscope. Scale bars 500nm.


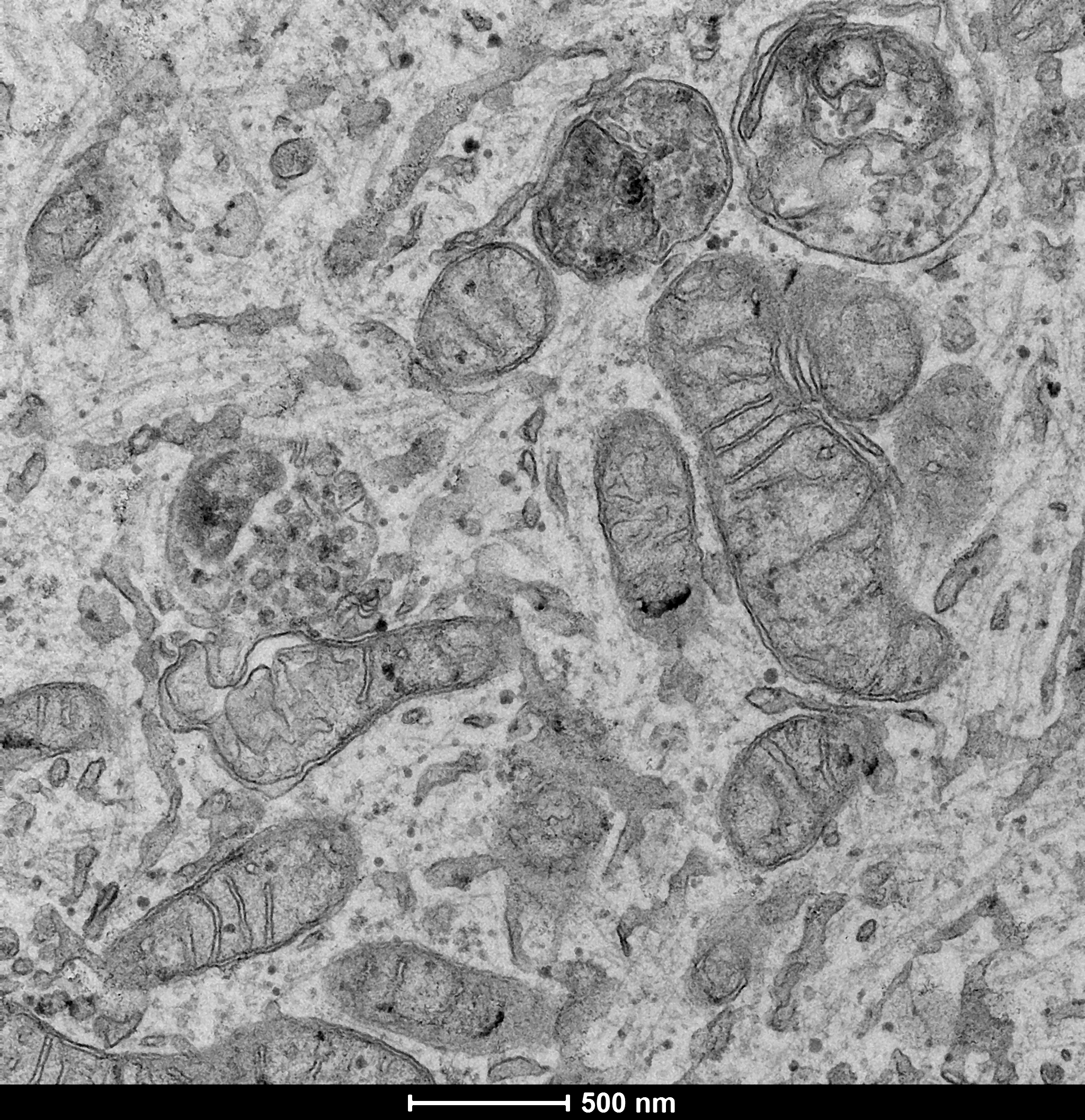

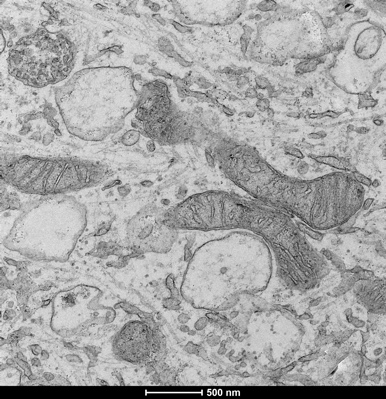


**Control 1**

**PKAN_[Tyr190*]_**


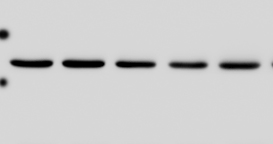

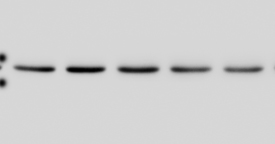


**EAAT2**

**Actin**

**Supplementary Figure 3. Uncropped western blots**


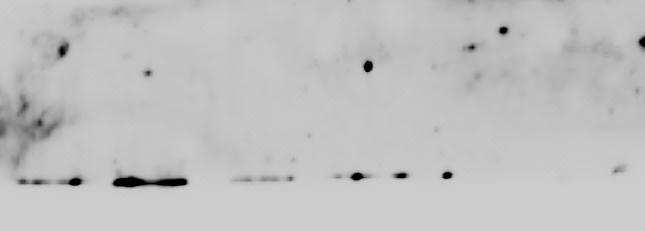

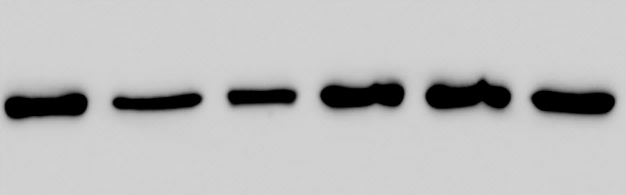


**NCOA4**

**Actin**


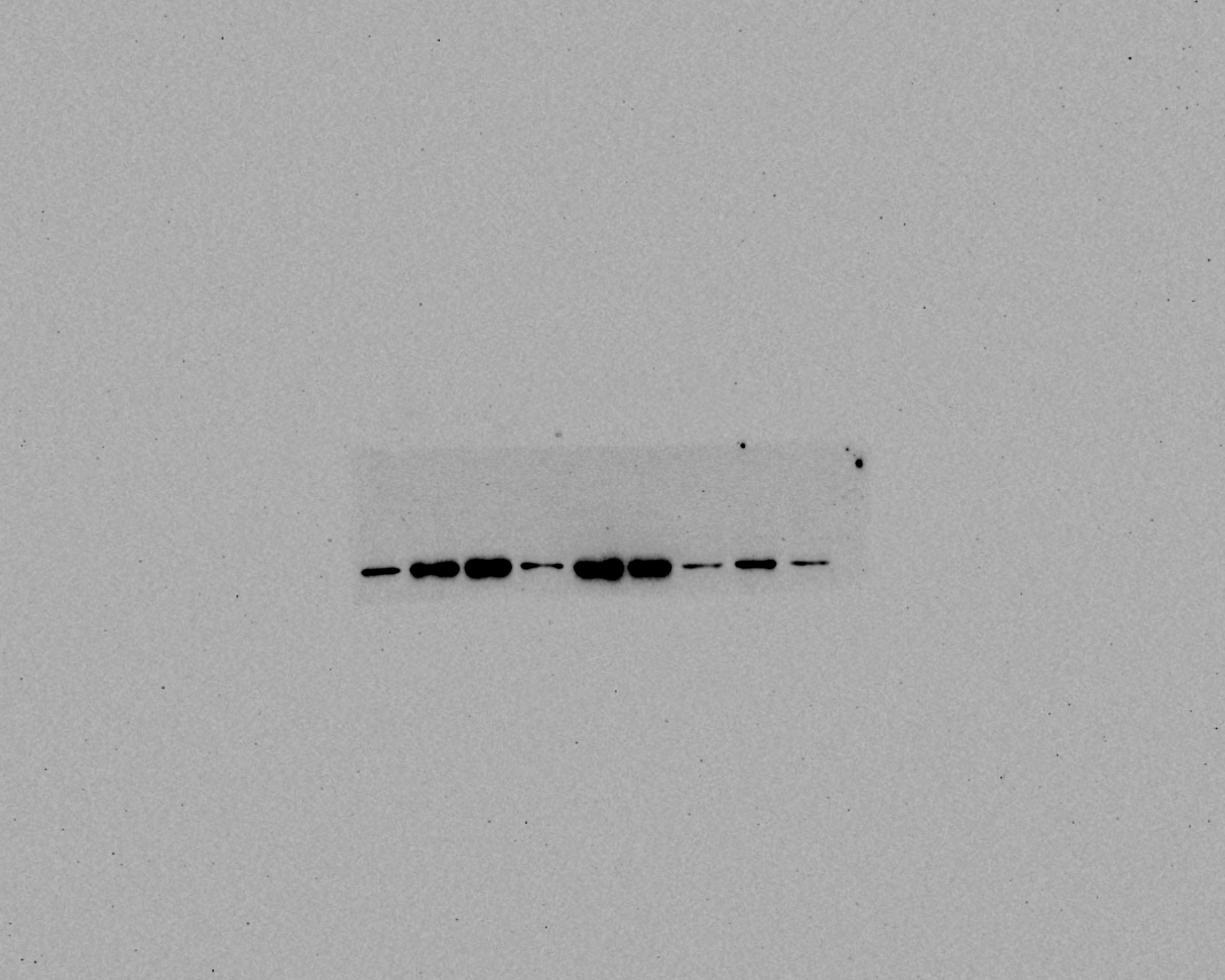


**DMT1 (IRE)**

**Actin**


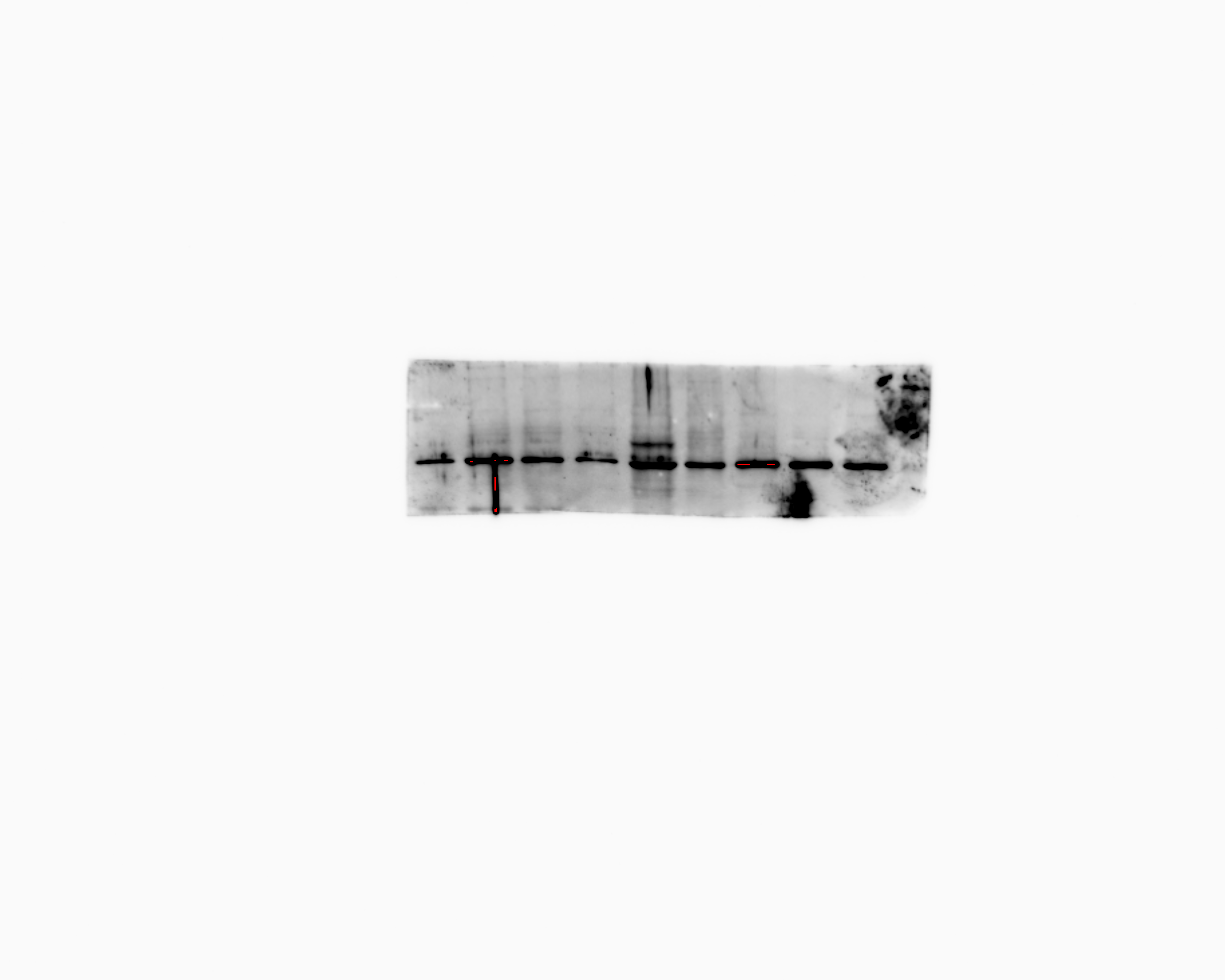

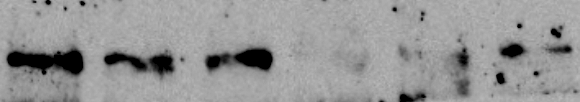

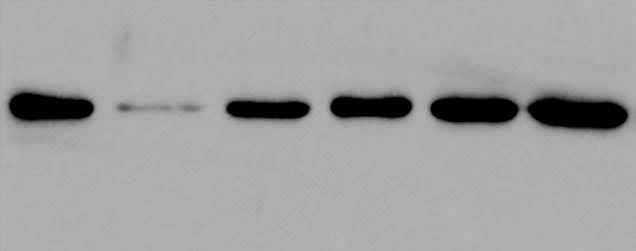


**TfR1**

**Actin**


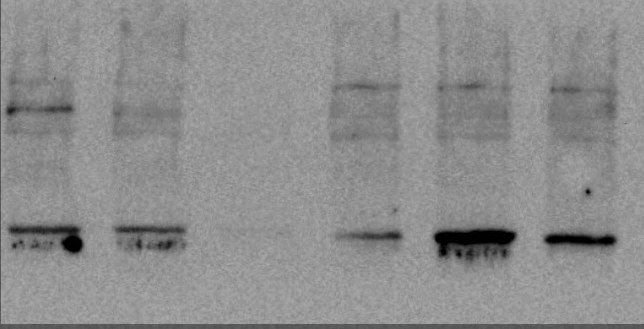

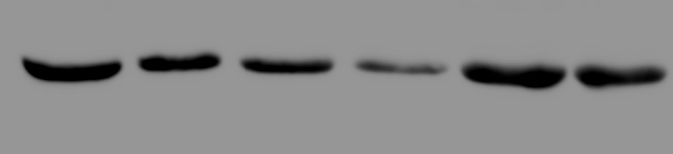


**FPN**

**Actin**


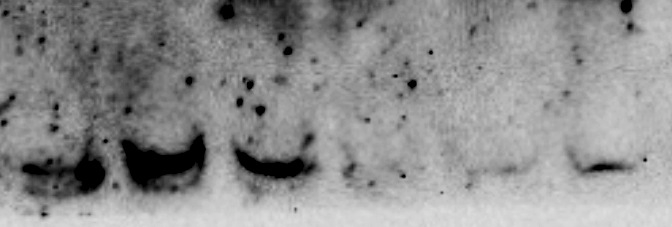

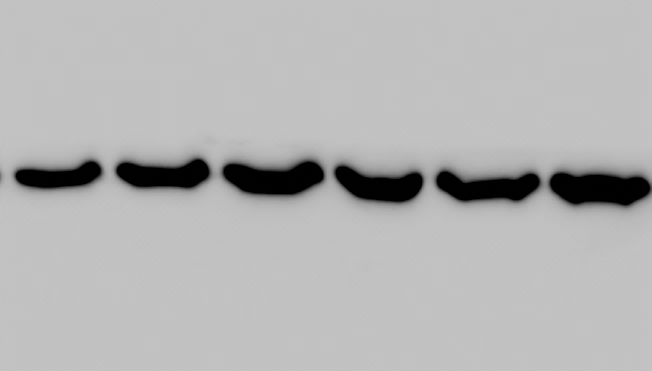


**GPX4**

**Actin**


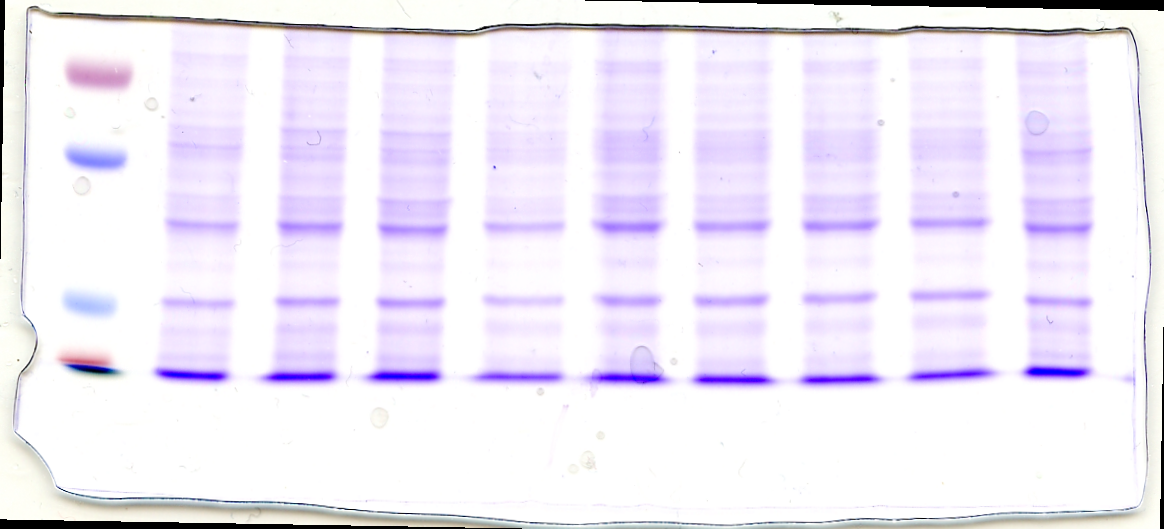

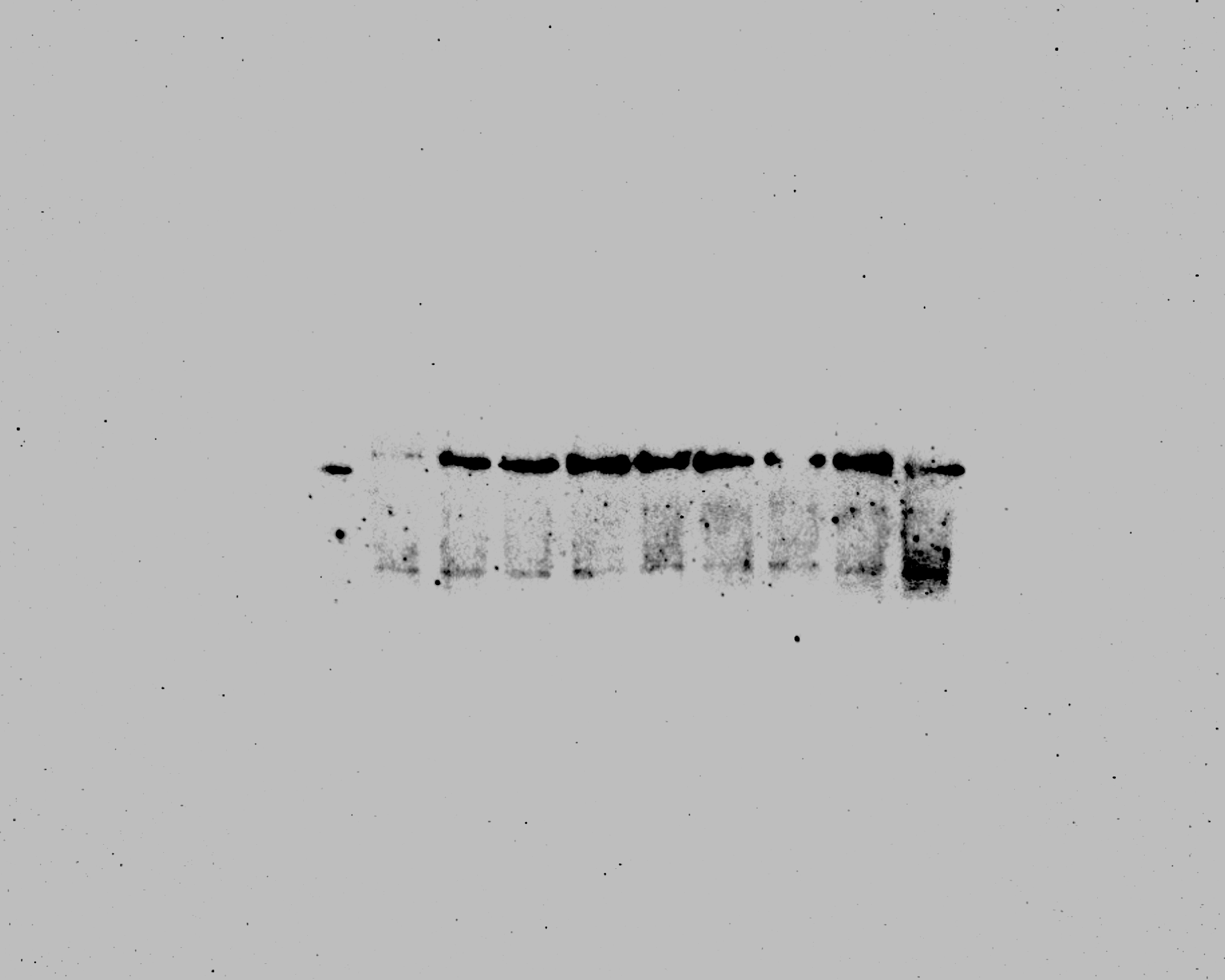


**Coomassie**

**Blue**

**FtH**

**Supplementary Table I. Antibodies used in immunofluorescence and immunoblotting studies.**

| **Antibody-Species^a^** | **Manufacturer** | **Catalog Number** | **Application ^b^ and Dilution** |
| --- | --- | --- | --- |
| Nestin - Ms | Millipore | MAB5326 | IF 1:500 |
| SOX2 - Rb | Abcam | AB59776 | IF 1:250 |
| Pax6 - Rb | Covance | PRB278P | IF 1:200 |
| MAP2 – Ms | Imm. Sciences | MAB10334 | IF 1:400 |
| MAP2 – Rb | Imm. Sciences | AB5622 | IF 1:400 |
| GAD65/67 - Rb | Sigma–Aldrich | G5163 | IF 1:200 |
| DARPP32 - Rb | Covance | AB1656 | IF 1:500 |
| TH - Rb | Covance | AB10312 | IF 1:200 |
| GABA – Ms | Sigma–Aldrich | A2052 | IF 1:200 |
| V-glut1 - Gp | Synaptic System | 135304 | IF 1:200 |
| GFAP - Ms | Millipore | MAB360 | IF 1:250 |
| EAAT2 - Ms | Santa Cruz Biotech. | SC-365634 | IF 1:200  WB 1:500 |
| β-tubulin - Ms | Sigma–Aldrich | T8328 | IF 1:300 |
| FtH - Ms | Home made | Luzzago et al., 1986 | IF 1:2 000  WB 1:500 |
| LAMP1 - Rb | Sigma–Aldrich | L1418 | IF 1:300 |
| β-actin - Ms | Sigma–Aldrich | A5441 | WB 1:6 000 |
| TfR1 - Mo | Zymed Laboratories | 13-6800 | WB 1:1 500 |
| FPN - Rb | Alpha Diagnostic | MTP11-S | WB 1:1 000 |
| DMT1-IRE - Rb | Alpha Diagnostic | NRAMP22-S | WB 1:1 000 |
| MDA - Rb | Cell Biolabs | 233101 | WB 1:800 |
| NCOA4 - Rb | Santa Cruz Biotech. | SC-28749 | WB 1:500 |
| GPX4 - Rb | ABclonal | A13309 | WB 1:2 000 |
| Mouse IgG Alexa488 - Dk | Imm. Sciences | IS20014 | IF 1:800 |
| Mouse IgG Alexa546 - Dk | Imm. Sciences | IS20305 | IF 1:800 |
| Rabbit IgG Alexa488 - Dk | Imm. Sciences | IS20015 | IF 1:800 |
| Rabbit IgG Alexa546 - Dk | Imm. Sciences | IS20308 | IF 1:800 |
| Guinea pig IgG Alexa594 - Gt | Mol. Probes | A11076 | IF 1:800 |
| Mouse IgG HRP - Rb | Sigma–Aldrich | A9044 | WB 1:100 000 |
| Rabbit IgG HRP - Gt | Sigma–Aldrich | A9169 | WB 1:100 000 |

^a^ Ms=mouse, Rb=rabbit, Gp=guinea pig, Dk=donkey, Gt=goat.

^b^ IF=immunofluorescence, WB=Western blotting (immunoblotting)
