# Supplementary material for: Massive iron accumulation in PKAN-derived neurons and astrocytes: light on the human pathological phenotype
Source: Cell Death Dis. 2022 Feb 25;13(2):185. doi: 10.1038/s41419-022-04626-x (PMC8881507; doi:10.1038/s41419-022-04626-x)
Supplement: Supplementary file 2 — English Editing Certificate [file 41419_2022_4626_MOESM2_ESM.pdf]

Editing Certificate

This document certifies that the manuscript

Massive iron accumulation in PKAN derived neurons and astrocytes: light on the human pathological phenotype.

prepared by the authors

Paolo Santambrogio1, Maddalena Ripamonti2, Anna Cozzi1, Marzia Raimondi2, Chiara Cavestro3, Ivano Di Meo3, Alicia Rubio1,4, Stefano Taverna1, Valeria Tiranti3* and Sonia Levi1,2*.

was edited for proper English language, grammar, punctuation, spelling, and overall style by one or more of the highly qualified native English speaking editors at AJE.

This certificate was issued on **December 21, 2021** and may be verified on the AJE website using the verification code **B9B0-7510-37F7-0F20-03E2**.

Neither the research content nor the authors' intentions were altered in any way during the editing process. Documents receiving this certification should be English-ready for publication; however, the author has the ability to accept or reject our suggestions and changes. To verify the final AJE edited version, please visit our verification page at aje.com/certificate. If you have any questions or concerns about this edited document, please contact AJE at support@aje.com.

AJE provides a range of editing, translation, and manuscript services for researchers and publishers around the world. For more information about our company, services, and partner discounts, please visit aje.com.
